# Grateful Acknowledgement

**Published:** 1887-05

**Authors:** F. E. Daniel


### GRATEFUL ACKNOWLEDGMENT.

"Our real existence hath truly its springs
Less in that which we do, than in that which we feel."

It was Shakespeare who said, "Friendship's but a name."

Poor Shakespeare! With his heaven-born genius—his heaven-sent immortality, he was to be pitied! He never felt within his own bosom, the thrill of that purest and most ennobling of all emotions vouchsafed to the human heart, nor experienced in his life, its vivifying, cheering, delightful influence, shed upon him by his kind !

Friendship is *not* a name: it is an entity;—it is real,—real as Love,—strong as Fate,—enduring as Time. It ends only at the grave, and hallowed memories then take its place. It is a compound sentiment, born of Respect,—and comprehends Faith, Trust, Esteem. It is a quality of the heart, which, like the perfume of the violet, sweetest when crushed—shines brighter in adversity: or, like the glow-worm's light, gleams brightest in the darkness of danger. It "comes out strong," as Mark Tapley would say, when least expected and—most needed !

To feel and realize that one has friends who will dare death in ones defense,—or, suffer sacrifice;—will assert their friendship warm—generous, enduring;—unselfish, disinterested;—twin brother of Love and Faith,—at a time of one's adversity or peril—ennobles and lifts up man's nature. It awakens sentiments strange, and new, and delightful; it invests life with new interests,—new beauties; and strengthens the ties that bind to earth,—making Heaven, e'en less desirable ! How delightful, when kindred souls strike hands, as it were,—and divining each other by intuition, read in the heart "Sincerity," "Trust," "Truth;" and should a cloud of mistrust fall athwart the bright horizon, obscuring those characters; should suspicion, serpent-like, creep in, or prejudice usurp the place of "Trust,"—how glorious when the sunlight of Reason, and Explanation, dispels the mists, and they rise up and clear away—leaving all brighter,—clearer, holier than before; like a mirror tarnished by a passing breath.

All this, and more, we felt, when, in an hour of sore trial,—of bitter, cruel, relentless persecution, the glorious light of friendship sprung from a thousand warm hearts—some, before not known or suspected,—and shed its cheering rays upon us,—sustaining and bidding us hope, when all was dark and drear! Words—poor words,—fail to express the emotions which fill the heart, or to translate the thoughts that crowd the mind,—when we recall that hour! We can only say to each and all—"God bless you!"—and "*never*, till life and memory perish, can I forget how dear thou art to me."

F. E. Daniel.

